# A systematic methodology to capture the global pattern of rheumatic heart disease: the Rheumatic Heart Disease Endemicity Index (RHDEI)

**DOI:** 10.1186/s44263-025-00179-1

**Published:** 2025-07-14

**Authors:** Megan Lindstrom, Neema W. Minja, Benjamin Stark, Catherine O. Johnson, Christian Razo, Nicole DeCleene, Kate E. LeGrand, George Mensah, David Watkins, Gregory A. Roth

**Affiliations:** 1 Institute for Health Metrics and Evaluation, University of Washington, Seattle, WA USA; 2 Department of Global Health, University of Washington, Seattle, WA USA; 3 Kilimanjaro Clinical Research Institute, Moshi, Tanzania; 4 Department of Health Metrics Sciences, University of Washington, Seattle, WA USA; 5 National Heart, Lung, and Blood Institute, National Institutes of Health, Bethesda, MD USA; 6 Department of Medicine, University of Washington, Seattle, WA USA; 7 Division of Cardiology, Medicine, University of Washington, Seattle, WA USA

**Keywords:** Rheumatic heart disease, Disease burden, Endemicity

## Abstract

**Background:**

Rheumatic heart disease (RHD) disproportionally affects young populations in socio-economically disadvantaged settings, resulting in a skewed distribution towards low- and middle-income countries. There is currently no consistent global surveillance system to identify countries with a high risk of RHD, which is a major barrier to addressing this public health threat. This paper describes a new methodology for conceptualizing locations at risk for high RHD morbidity and mortality, or burden, globally.

**Methods:**

We utilized a set of covariates produced by the Global Burden of Disease Study from 1990 to 2021 via principal component analysis to create the rheumatic heart disease endemicity index (RHDEI). We then demonstrate how the RHDEI could be used in forecasting for targeted policy change with the use of an ensemble time-series forecasting model, creating 20 years of estimates through 2041. The results were evaluated via out-of-sample forecasting to estimate model performance and compared to a naive model to assess goodness of fit.

**Results:**

We produced 203 country-level yearly estimates from 1990 to 2021 for the RHDEI. We found that countries in sub-Saharan Africa and South-East Asia had the highest RHDEI results, reflecting the burden in those regions. The largest decrease in RHDEI was estimated for South Sudan, and the largest increase was estimated for Angola. Our forecast through 2041 further highlighted the heterogeneity of RHD burden, demonstrating how without intervention some regions will likely see worse outcomes in relation to RHD.

**Conclusion:**

The RHDEI provides a much-needed method for capturing global RHD distributions that can improve our understanding of the changing patterns in a data scarce landscape. The evidence the index provides can help researchers, policy makers, and clinicians better understand RHD burden and act to reduce it.

**Supplementary Information:**

The online version contains supplementary material available at 10.1186/s44263-025-00179-1.

## Background

In 2022, rheumatic heart disease (RHD) affected over 55 million people worldwide, the majority in low- and middle-income countries (LMIC) [1]. RHD is the result of repeated episodes of acute rheumatic fever (ARF), an autoimmune reaction to a throat infection by Group A *Streptococcus* (GAS) in predisposed individuals [1, 2]. Despite being preventable, RHD persists in many parts of the world and is the most significant acquired cardiovascular disease in adolescents and young adults globally, claiming 386,947 lives across all ages in 2022 [1].

RHD is a disease of poverty in which underlying health disparities and socio-economic drivers result in a distinct disease pattern globally, largely limited to LMICs and minority populations in low-resource settings within high-income countries [3, 4]. The compounded impact in regions most affected are large, with the median age at death found to be 28 years in sub-Saharan African (SSA) and Asian countries, portending substantial economic consequences [5]. Between 1990 and 2019, age-standardized incidence and prevalence rates of RHD have increased by 14.4% (11.2–17.0%) and 13.8% (11.0-–16.0%), respectively, with this increase limited to low- and low-middle sociodemographic index (SDI) locations, while high-middle and high SDI locations showed a decreasing trend [6]. Several studies have demonstrated the association between social determinants of health (SDH) and RHD, revealing a positive association with various factors including overcrowding, poor socio-economic status, low education levels, unemployment, poor access to healthcare, dwelling characteristics, and poor hygiene [3, 6–8]. Once common in the mid- to late twentieth century in the high-income developed world, RHD has shown a steep decline in many high-income countries and other rapidly growing economies that have seen parallel improvements in living conditions and healthcare or have invested in intensive, targeted interventions [9–11]. In contrast, many challenges still exist to effectively control RHD programs in regions most affected, encompassing both socio-economic determinants of health and health system challenges that largely define its current geographical distribution.

Contemporary evidence-based RHD control efforts focus on prevention at different levels of disease progression. Primordial prevention involves the modification of SDH that predisposes populations to GAS infections and its subsequent progression to RHD [12]. The National Heart, Lung, and Blood Institute (NHLBI) identified several areas for further research to inform policy and interventions in the primordial prevention of RHD. One of the key areas emphasized was “to assess and map the distribution of GAS infection, ARF incidence and RHD prevalence in countries and geographical regions in relation to SDH and learn from their experience [13].” Despite these calls for research, there has continued to be a significant lack of systematic, global data to support interventions.

Due to the impact of SDH, RHD is considered an endemic disease linked to certain locations and populations across time. However, the lack of systematic surveillance complicates defining which locations should be considered endemic or non-endemic. This scarcity of data makes much needed evidence-based policy and prevention programs difficult to implement, particularly in LMICs where the largest burden is seen. In the absence of active national surveillance systems, we propose an update to the next best methodological approach of defining RHD burden globally, providing a comprehensive and robust classification scheme that addresses the need for data that can support RHD policy and interventions. We demonstrate a novel method that combines the known underlying drivers of RHD into the rheumatic heart disease endemicity index (RHDEI) via principal component analysis (PCA) for each year from 1990–2021. We then demonstrate how the index could be used to help policy implementation in a case study focused on forecasting.

## Methods

We created an index, termed the RHDEI, to determine RHD burden and track patterns and trends over time globally. We identified known drivers of RHD burden from literature and expert consultation, matching these variables to data available from the Global Burden of Disease (GBD) and employing PCA to create the final index. We then forecasted the index with an ensemble time-series model to demonstrate how the index could be used to understand the potential future global landscape of RHD. We produced the RHDEI by GBD super region, defined as seven global regions grouped by epidemiological and geographic similarities (Additional File 1: Fig. S1). Our study follows the Guidelines for Accurate and Transparent Health Estimates Reporting (GATHER (Additional File 1: Table S1)). All statistical analyses and visualizations were performed and created in R statistical computing language (Version 4.4.0, Vienna, Austria) [25].

The initial step in the creation of the RHDEI was the identification of the disease’s underlying drivers. Frequently documented factors included overcrowding, education level, dwelling characteristics, poor hygiene, access to healthcare, unemployment, and undernutrition [3, 7, 14, 15]. Coffey et al. highlighted that there is strong evidence towards a causative relationship between overcrowding and RHD precursors with a demonstratable continuous effect gradient [3]. This factor was postulated to play a catalytic role between poverty and RHD burden in affected countries. Other factors, including maternal employment and education level, were also found to be associated with ARF/RHD in several studies, and this association remained significant in multivariable analyses but was not universally consistent [16–18]. In Uganda, access to healthcare, measured as the physical distance from health services, was a significant factor towards RHD case outcomes [7, 19]. Further, studies on nutrition found an association between undernutrition and ARF or RHD using weight-based measures [3]. For several of these factors, although results of studies have been heterogeneous and differed in design, population, and overall quality, a positive association with a gradient has been demonstrated for socio-economic status and GAS infection, ARF, and RHD risk [3, 20, 21].

These factors were cross-referenced with variables created for the study to identify the most important covariates to include in the RHDEI [22]. We chose to include the following GBD covariates as they provide consistent spatiotemporal granularity: the sociodemographic index (SDI), the healthcare access and quality index (HAQI), proportion of population with access to improved sanitation, proportion of population with access to improved water sources, proportion of children in the population underweight, and all-cause mortality rate for the population aged 10–14. Each of these variables was retrieved for the years 1990–2021 for 813 global locations made available by GBD. While some of these variables are not direct measurements of the drivers identified in the literature, they sufficiently capture the factors identified in the literature.

We then performed a PCA, a dimensionality reduction method that takes a dataset of variables for *n* entities (subjects, locations, etc.) and identifies the linear combinations that result in the highest variance via orthogonal lines of best fit [23]. We were primarily interested in the first component which captures the greatest amount of variance in the dataset and can be used as a composite index. This method was selected for the creation of the RHDEI because it provided a single measure based on correlated variables which is much simpler to interpret and apply to subsequent methodological steps. The basic equation for the PCA computation is as follows [24]:

$${c}_{1}={\beta }_{1p}{x}_{1}+{\beta }_{12}{x}_{2}+...+{\beta }_{1p}{x}_{p}$$

where ${c}_{1}$ is the entity’s principal component score for the first component, ${\beta }_{1p}$ is the weight for observed variable $p$, and ${x}_{p}$ is the value of the observed variable $p$ for that entity. This equation is calculated the same number of times as there are variables in the dataset, resulting in multiple $c$’s. The PCA was completed in R, utilizing the prcomp() command [25]. We used the scaling and centering options provided by the function as PCA is affected by scale. In addition, variables for all-cause death and underweight were inverted so that the direction of all variables would uniformly indicate better health outcomes with increasing values (i.e., locations with higher values of each variable would have lower rates of the drivers associated with RHD).

The final step was to forecast the index with an ensemble time-series model. We used the forecastHybrid modeling package in R to demonstrate the application of the RHDEI [26]. This package is a time-series forecasting modeling tool that has been utilized by researchers across multiple sectors including public health and climate that allow for the specification of a set of forecasting models combined via a weighting scheme into a final ensemble model [27, 28]. More specifically, in its default setting, the model attempts to fit a combination of an autoregressive integrated moving average (auto.arima) model, exponential smoothing state space (ETS) model, theta model (Thetam), neural network time series (Nnetar) model, seasonal and trend decomposition using loess (STLM) model, and an exponential smoothing state space model with Box-Cox transformation, autoregressive moving average (ARMA) errors, and trend and seasonal components (TBATS) model. Once it selects the models, it weights them to create a combined mean estimate of forecasts. There are multiple options for weighting the models, and we employed with the default of equal weighting which has been shown in studies to produce statistically robust results [27, 29]. This method is well suited for use cases where many iterations of the forecast must be run without fine-tuning of each individual model due to the aggregation of the selected forecasting models. Results may be more conservative with wider uncertainty, due to their being an equally weighted average of the final selected models, which can be a useful feature when used for forecasting future trends.

For this case study, we ran an ensemble model that included all forecasting submodels, except for the ETS and STLM due to this being non-seasonal yearly data, for inclusion in the final equal weighted mean model (each model receiving a weight of 0.25). TBATS was inspected and ultimately included as it was not applying a seasonal effect to the data. The ensemble model was iterated over each location’s RHDEI data from 1990 to 2021 individually and then produced a forecast for the next 20 years based on these data. The original RHDEI and forecasted data were then merged into a single dataset containing all years and locations of RHDEI data from 1990 to 2041. To ensure that the forecasts stayed within the bounds of the index (0, 1), the data was first transformed using a scaled logit transformation and then transformed back for use and interpretation. We cross-validated our model results using the cvts() function from the forecastHybrid R package, which uses out-of-sample forecasting to estimate model performance. We compared the root-mean-square error (RMSE) of our hybrid model with that of a naive model to determine goodness of fit.

The full output of the analyses and details about data sources are publicly available in the Global Health Data Exchange (https://ghdx.healthdata.org/record/ihme-data/global-rhdei-estimates-forecasts-1990-2041).

## Results

### RHDEI

Descriptive statistics for 1990 and 2021, including the mean, minimum, and maximum values for variables included in the PCA and grouped by GBD super region (with the number of locations found within the super region in parentheses), are shown in Table 1. All super regions measured an improvement in each of the variables, showing an overall global trend towards the improvement of underlying RHD-related drivers.

**Table 1:** Mean and range for variables included in the principal component analysis (PCA) for each super region, 1990 and 2021

**Super region**	**Year**	Mean (min, max)					
		**SDI**	**HAQI**	**Access to sanitation**	**Access to improved water**	**Proportion of children underweight**	**Mortality rate per 100,000 for ages 10–14**
Central Europe, Eastern Europe, and Central Asia (n=112)	1990	0.64 (0.47, 0.82)	52.87 (25.51, 67.88)	0.91 (0.57, 1.00)	0.96 (0.52, 1.00)	0.02 (0.01, 0.09)	43.40 (21.74, 78.71)
	2021	0.78 (0.54, 0.89)	69.32 (42.94, 89.23)	0.96 (0.80, 1.00)	0.99 (0.81, 1.00)	0.01 (0.01, 0.05)	22.02 (7.59, 43.09)
High income (n=181)	1990	0.75 (0.58, 0.87)	73.56 (43.90, 82.20)	0.98 (0.70, 1.00)	1.00 (0.97, 1.00)	0.02 (0.01, 0.07)	21.52 (9.44, 89.86)
	2021	0.85 (0.72, 0.95)	86.55 (58.68, 94.23)	0.99 (0.97, 1.00)	1.00 (1.00, 1.00)	0.01 (0.01, 0.05)	11.01 (5.48, 38.91)
Latin America and Caribbean (n=92)	1990	0.49 (0.31, 0.70)	36.65 (11.01, 56.39)	0.70 (0.39, 0.99)	0.83 (0.46, 0.99)	0.04 (0.01, 0.16)	53.08 (16.90, 129.13)
	2021	0.65 (0.45, 0.83)	53.85 (22.98, 80.02)	0.90 (0.59, 0.99)	0.97 (0.75, 1.00)	0.02 (0.01, 0.07)	32.07 (7.83, 76.29)
North Africa and Middle East (n=52)	1990	0.44 (0.17, 0.66)	40.30 (14.53, 61.74)	0.56 (0.24, 0.95)	0.89 (0.48, 0.99)	0.07 (0.02, 0.23)	117.57 (41.13, 1238.39)
	2021	0.68 (0.34, 0.85)	64.17 (29.35, 78.83)	0.91 (0.64, 1.00)	0.98 (0.72, 1.00)	0.03 (0.01, 0.18)	30.47 (13.28, 66.96)
South Asia (n=43)	1990	0.34 (0.20, 0.52)	24.05 (17.31, 38.35)	0.36 (0.13, 0.73)	0.66 (0.47, 0.90)	0.24 (0.10, 0.40)	109.01 (52.04, 205.99)
	2021	0.57 (0.40, 0.73)	43.51 (31.00, 58.45)	0.76 (0.44, 0.99)	0.92 (0.80, 0.98)	0.13 (0.04, 0.24)	47.76 (23.63, 86.57)
Southeast Asia, East Asia, and Oceania (n=183)	1990	0.46 (0.24, 0.71)	32.56 (12.99, 61.82)	0.51 (0.12, 0.92)	0.61 (0.30, 0.98)	0.13 (0.01, 0.30)	78.99 (22.23, 240.89)
	2021	0.64 (0.42, 0.87)	48.60 (23.85, 86.27)	0.85 (0.36, 0.98)	0.90 (0.56, 1.00)	0.07 (0.01, 0.19)	39.96 (7.84, 79.57)
Sub-Saharan Africa (n=150)	1990	0.29 (0.05, 0.63)	23.54 (6.44, 40.05)	0.39 (0.10, 0.89)	0.48 (0.20, 0.99)	0.13 (0.04, 0.32)	113.20 (40.79, 326.08)
	2021	0.48 (0.08,0.74)	35.13 (14.30, 53.80)	0.56 (0.14, 0.94)	0.74 (0.40, 1.00)	0.08 (0.02, 0.22)	63.35 (30.48, 134.68)

*SDI* sociodemographic index, *HAQI* Healthcare Access and Quality Index

**Fig. 1:**
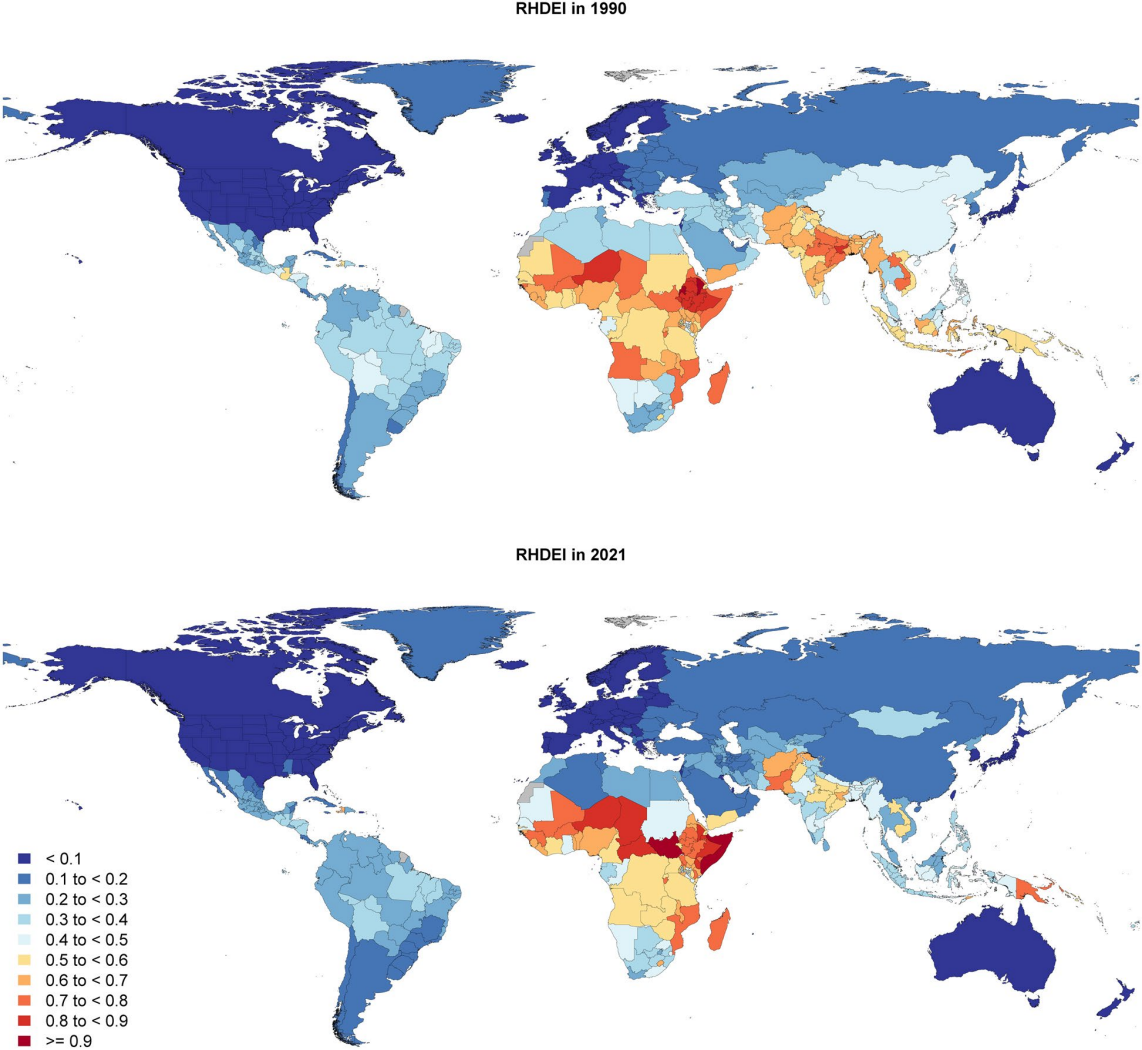
RHDEI in 1990 and 2021

In total, the PCA used data for 813 locations made available by the GBD, resulting in 203 country-level time-series estimates. The loadings for each of the principal components for both 1990 and 2021 can be found in Additional File 1: Table S2. We chose to extract only PC1 for the RHDEI, as it is the component that captures the most variance, accounting for 77% of the total variance in 1990 and 80% of the total variance in 2021. PC1 also has the only eigenvalue (square of standard deviation) greater than 1, further indicating that it sufficiently captures the variance to be utilized as a composite index of the selected variables. For 1990, the loadings for all the variables are between − 0.29 (all cause death) and − 0.45 (sociodemographic index). In 2021, the range tightens from − 0.36 (underweight) to − 0.44 (sociodemographic index). Overall, this indicates even loadings of the variables across the component. The final step of the RHDEI involved extracting the first principal component from the PCA and rescaling it from 0 to 1 for ease of interpretation. Figure 1 shows the results of the RHDEI for 1990 and 2021, with a number closer to zero corresponding to the location having lower rates of the variables positively associated with RHD. The locations with the highest values, or those that are more likely to have factors associated with higher rates of RHD, are demonstrated in sub-Saharan Africa and South-East Asia.

### Application of RHDEI in a forecasting case study

**Fig. 2:**
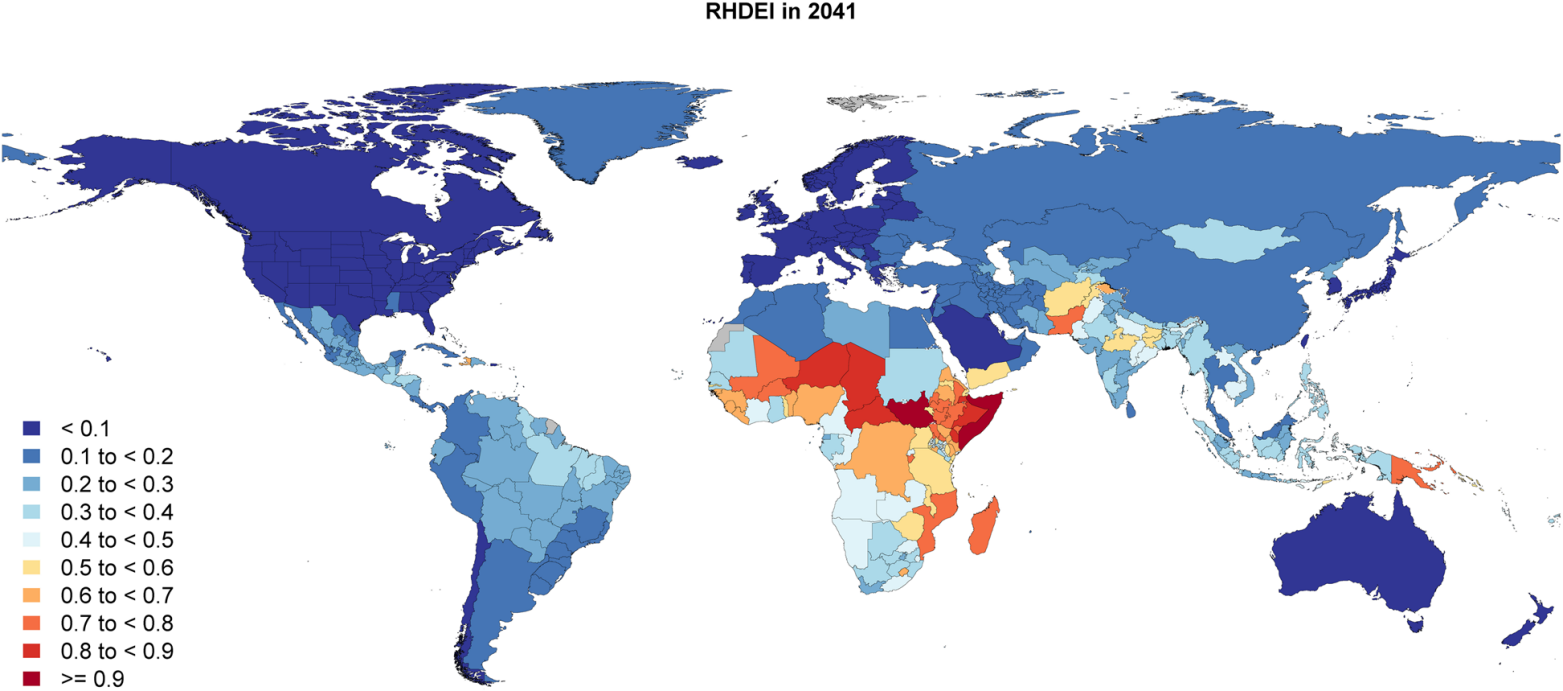
Forecasted RHDEI in 2041

To demonstrate the utility of the RHDEI, we utilized forecast modeling to project the RHDEI through 2041 (Fig. 2).

**Fig. 3:**
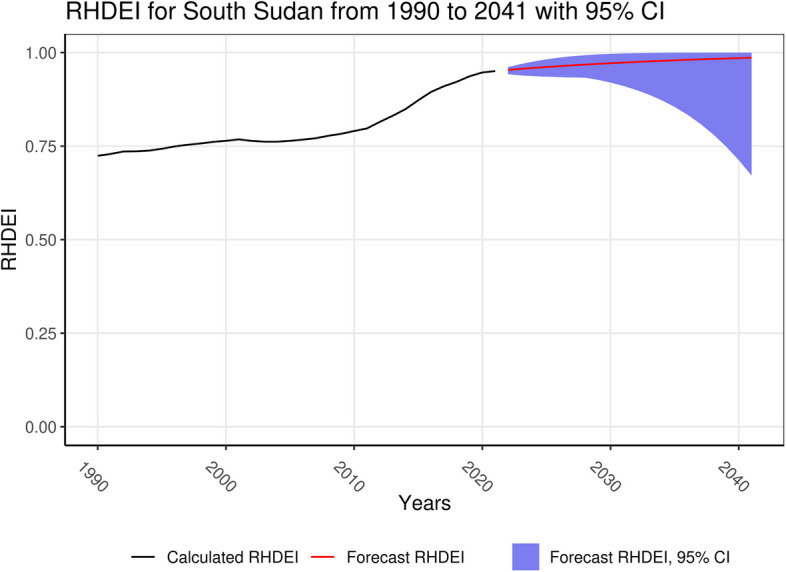
RHDEI for South Sudan with forecasted values to 2041 and 95% confidence intervals

**Fig. 4:**
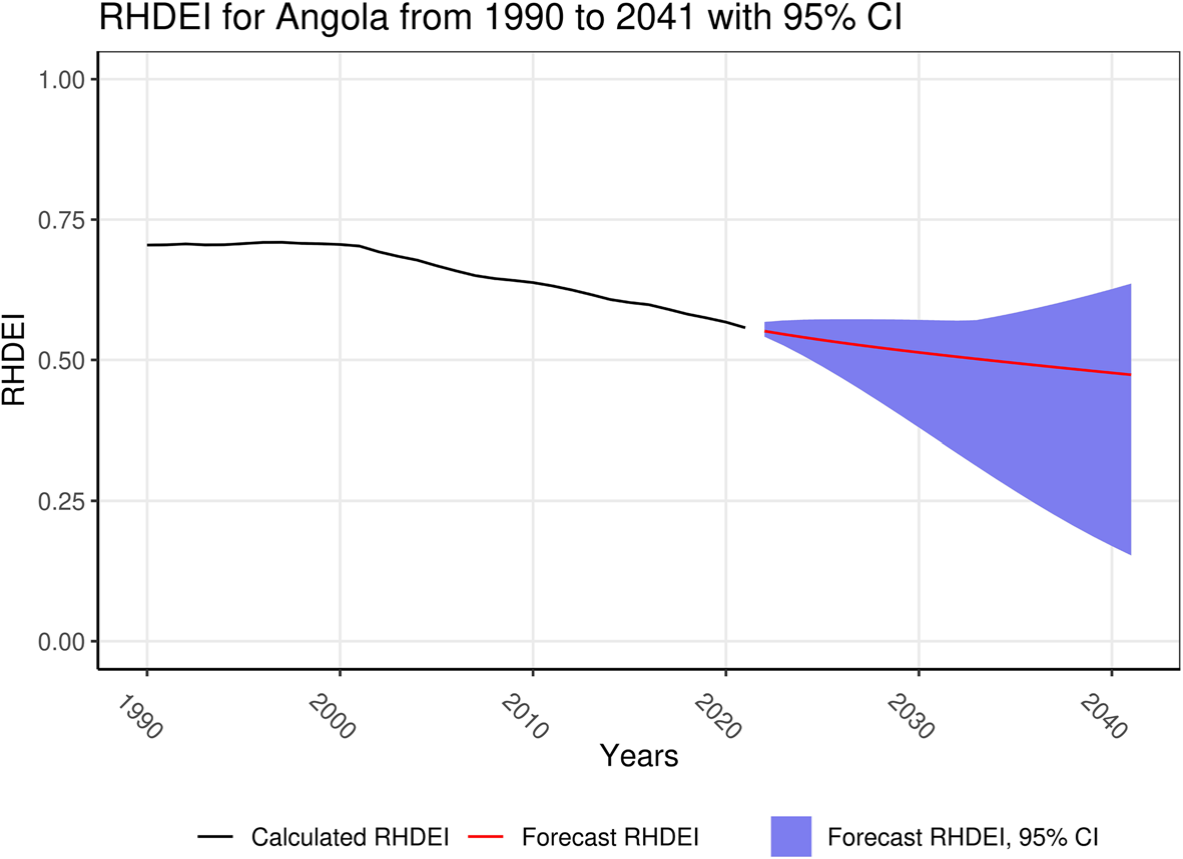
RHDEI for Angola with forecasted values to 2041 and 95% confidence intervals

Two countries with notable changes in RHDEI from 2021 to 2041 were South Sudan, seen in Fig. 3, with a 3.8% increase in the RHDEI when comparing 2041 to 2021, and Angola, seen in Fig. 4, with a 15% decrease in the RHDEI when comparing 2041 to 2021. This can be interpreted as meaning that based on the RHDEI estimates from 1990 to 2021 and with no further intervention, South Sudan could expect to see a continued increase in RHD burden through 2041, while Angola could expect to see a decrease in RHD burden through 2041.

During our cross-validation, we found the majority of locations (over 90%) had a better RMSE from the hybrid model versus the naive model (see Additional File 1: Fig. S2). We investigated locations that had a significantly worse RMSE from the hybrid model compared to the naive and found that this was most common in locations whose RHDEI was already close to the top or bottom of the index in 2021.

## Discussion

RHD is an important global health issue that is difficult to quantify due to data scarcity, particularly in areas with the highest burden. We describe a methodology to construct the RHDEI, an index that identifies global locations most at risk for high rates of RHD, across time. The RHDEI utilizes GBD variables from 1990 to 2021 associated with higher rates of RHD at the population level identified by literature. It combined SDI, HAQI, access to sanitation, access to improved water, proportion children underweight, and mortality rate for 10–14 years old per 100,000 via PCA for each year and location, providing a novel systematic quantification of the changing pattern of factors associated with RHD burden by country. We then demonstrated how the index can be utilized to forecast locations that may experience increased or decreased rates of risk factors associated with RHD and for use within the GBD modeling framework.

Through our forecasting case study, we highlighted how the RHDEI can be used to identify countries that may need tailored policies and interventions to reduce RHD or, conversely, are expected to see a substantial improvement in disease burden. Evidence indicates that significant reductions in ARF, and subsequently RHD, are possible through political commitment, policy, and targeted interventions. In Cuba, a 10-year program targeted at ARF/RHD was both cost-saving and successful in reducing the RHD burden [30, 31]. Experts on RHD in African regions, where the highest prevalence is recorded, have established a roadmap that prioritizes seven critical areas aimed at eliminating RHD. Specific policy recommendations include country-led RHD registries, prioritizing proven RHD interventions including secondary antibiotic prophylaxis, together with ensuring an adequate supply of medicines needed and, importantly, monitoring and assessment of disease burden [32]. Forecast modeling provides an avenue to address this priority and has become increasingly popular within the public health sphere, particularly with the COVID-19 pandemic [27, 33].

We highlighted two locations, South Sudan and Angola, in our results which could be examined further to help guide future health policy and funding decisions. The first location, South Sudan, has been heavily influenced by political instability — ongoing conflicts and war that translate to persistent impoverishment and further reductions in healthcare access largely dependent on humanitarian relief [34]. While Sudan in the North, which officially separated in 2011, also experiences conflict, it is better resourced with documented commitment and NGO support towards RHD control exemplified by the implementation of the “SUR I CAAN program (Surveillance, Integration, Communication, Awareness, Advocacy and Training)” [35]. The projected increase in RHDEI from the 2021 value signifies continued challenges expected over the next 20 years, underscoring the need for coordinated action through multi-sectoral programs and international partnerships to address ARF/RHD and other major contributors to poor health. Limited studies in Angola in the last decade reveal a substantial RHD burden while demonstrating the highest prevalence in areas with limited access to healthcare [36]. While published literature on RHD policy and implementation is scarce, we projected a decreased RHDEI in Angola. This signifies progress and could reflect improvements in socio-economic conditions consistent with a World Bank documented 3% increase in GDP over the past few years [37].

It is important to note that the RHDEI is not the first index calculated with the objective of assessing the geographical patterns of endemic diseases. More than six decades ago, the World Health Organization, created the “index of endemicity” from routinely collected national data on mortality, morbidity, and incidence rates [38]. This index was applied to various diseases including malaria, TB, and schistosomiasis; however, it has not been applied to RHD. In addition, our methodology is the first to use a PCA to calculate a global endemicity index for RHD. We now use the RHDEI as an input to modeling RHD burden statistics for the GBD.

A limitation of the RHDEI is the lack of direct validation methods. Global prevalence of RHD is only available from modeled results due to limited data availability, particularly in places we expect to see high RHD burden. Our analysis serves the immediate purpose to methodologically ascertain high burden areas and would greatly benefit from systematic screening programs in LMICs. Improved surveillance systems would provide a better data landscape that would allow for validation of the index. A second limitation lies in the forecasting method which uses equal weight combination of the forecasting models, though this has been noted as likely being the best approach in applications where many time series need to be run automatically [39]. A third limitation is that highly endemic populations within smaller subnational regions have not been estimated here. Future research could include extending this approach to the subnational level and investigating fine tuning of the weighting of forecasting models.

## Conclusions

RHD is a neglected disease of poverty that continues to pervade populations across the world. Though strides have been made to reduce morbidity and mortality in some locations, the burden remains significant in many LMICs. Continuous improvements to the global estimation methods for RHD are important to define the burden as accurately and systematically as possible with important implications for control efforts. This paper outlines a systematic methodology for quantifying RHD disease burden crucial to understanding RHD patterns geographically and transitions over time. The RHDEI provides a methodological robust conceptualization of RHD burden for every country and year — which can be used to support future research efforts and public health policy targeting the reduction of RHD globally.

## Supplementary Information

Additional file 1: Figure S1. Map of Global Burden of Disease super regions in 2021. Table S1. Guidelines for Accurate and Transparent Health Estimates Reporting (GATHER) checklist. Table S2. Principal component analysis factor loadings for 1990 and 2021. Figure S2. Comparison of cross-validated model root mean square error (RMSE).

## Data Availability

The findings of this study are supported by data available in public online repositories, data publicly available upon request of the data provider, and data not publicly available due to restrictions by the data provider. Non-publicly available data were used under license for the current study but may be available from the authors upon reasonable request and with permission of the data provider. Analytic code is publicly available from https://github.com/ihmeuw/rhdei [40]. Data are publicly available on the Global Health Data Exchange repository (GHDx), https://ghdx.healthdata.org/record/ihme-data/global-rhdei-estimates-forecasts-1990-2041[41].

## Abbreviations

RHD: Rheumatic heart disease
LMICs: Low- and middle-income countries
ARF: Acute rheumatic fever
GAS: Group A *Streptococcus*
SSA: Sub-Saharan Africa
SDI: Sociodemographic index
SDH: Social determinants of health
NHLBI: National Heart, Lung, and Blood Institute
RHDEI: Rheumatic heart disease endemicity index
GBD: Global burden of disease
HAQI: Health Access and Quality Index
PCA: Principal component analysis
ETS: Exponential smoothing state
Thetam: Theta model
Nnetar: Neural network time series
STLM: Seasonal and trend decomposition using loess
TBATS: Trend and seasonal components
