# Supplementary material for: A systematic methodology to capture the global pattern of rheumatic heart disease: the Rheumatic Heart Disease Endemicity Index (RHDEI)
Source: BMC Glob Public Health. 2025 Jul 14;3:62. doi: 10.1186/s44263-025-00179-1 (PMC12261633; doi:10.1186/s44263-025-00179-1)
Supplement: Supplementary file 1 — Additional file 1: Figure S1. Map of Global Burden of Disease super regions in 2021. Table S1. Guidelines for Accurate and Transparent Health Estimates Reporting (GATHER) checklist. Table S2. Principal component analysis factor loadings for 1990 and 2021. Figure S2. Comparison of cross-validated model root mean square error (RMSE). [file 44263_2025_179_MOESM1_ESM.docx]

**A systematic methodology to capture the global pattern of Rheumatic Heart Disease: The Rheumatic Heart Disease Endemicity Index (RHDEI)**

**ADDITIONAL FILE 1**

**Contents**

[Figure S1. Map of Global Burden of Disease super regions in 2021 2](#_Toc1191713239)

[Table S1. Guidelines for Accurate and Transparent Health Estimates Reporting (GATHER) checklist 2](#_Toc542019888)

[Table S2. Principal component analysis factor loadings for 1990 and 2021 4](#_Toc780962627)

[Figure S2. Comparison of cross-validated model root mean square error (RMSE) 6](#_Toc763979289)

We produced the Rheumatic Heart Disease Endemicity Index, grouped by each Global Burden of Disease super region. These groupings are defined by epidemiological and geographic similarities and presented in supplemental figure S1. The full list of locations in the Global Burden of Disease hierarchy is available from <https://www.healthdata.org/research-analysis/about-gbd/gbd-data-and-tools-guide>.


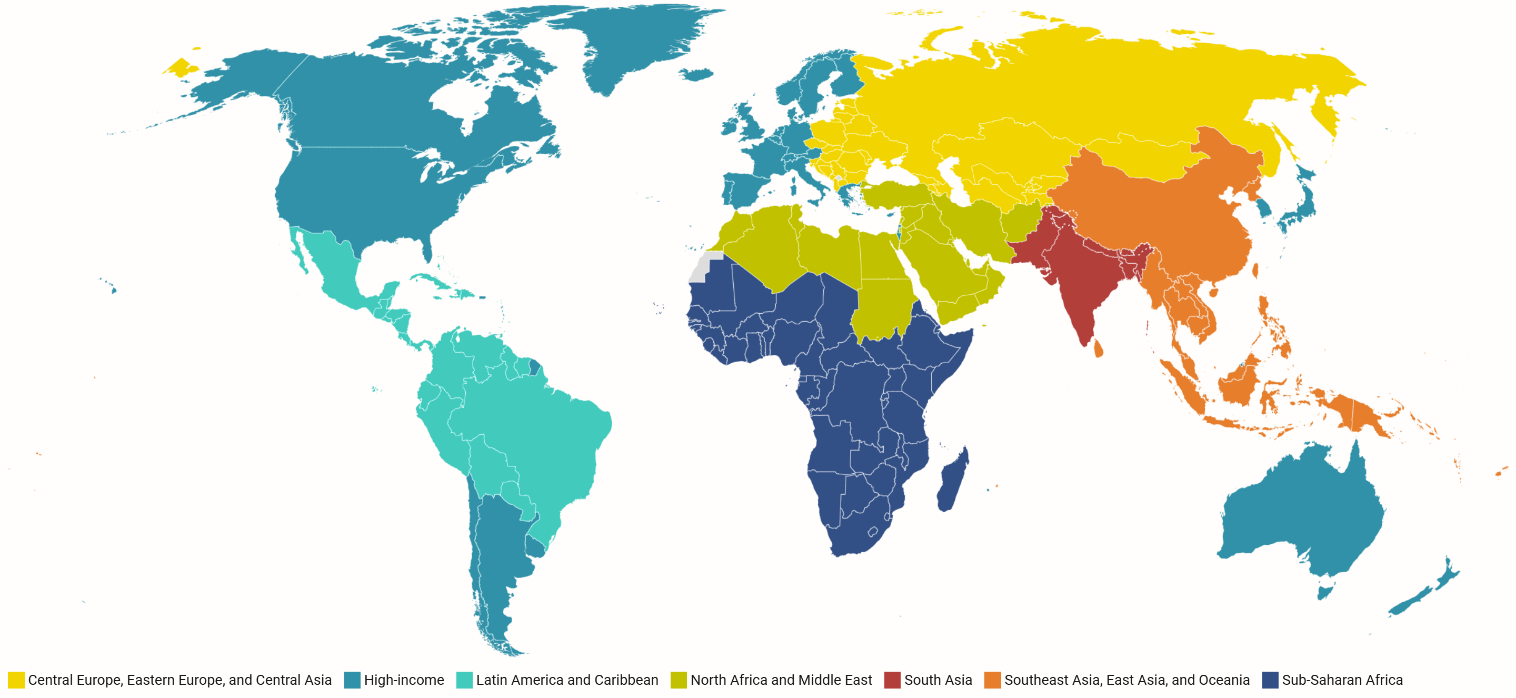


# Figure S1. Map of Global Burden of Disease super regions in 2021

# Table S1. Guidelines for Accurate and Transparent Health Estimates Reporting (GATHER) checklist

| **Item** | **Checklist item** | **Reported on page #** |
| --- | --- | --- |
| **Objectives and funding** | | |
| **1** | Define the indicator(s), populations (including age, sex, and geographic entities), and  time period(s) for which estimates were made. | 6 |
| **2** | List the funding sources for the work. | 13 |
| **Data Inputs** | | |
| *For all data inputs from multiple sources that are synthesized as part of the study:* | | |
| **3** | Describe how the data were identified and how the data were accessed. | 13 |
| **4** | Specify the inclusion and exclusion criteria. Identify all ad‐hoc exclusions. | 13 |
| **5** | Provide information on all included data sources and their main characteristics. For each data source used, report reference information or contact name/institution, population represented, data collection method, year(s) of data collection, sex and age range, diagnostic criteria or measurement method, and sample size, as relevant. | 13, Global Health Data Exchange (GHDx) https://ghdx.healthdata.org/record/ihme-data/rhdei |
| **6** | Identify and describe any categories of input data that have potentially important biases (e.g., based on characteristics listed in item 5). | 13 |
| *For data inputs that contribute to the analysis but were not synthesized as part of the study:* | | |
| **7** | Describe and give sources for any other data inputs. | N/A |
| *For all data inputs:* | | |
| **8** | Provide all data inputs in a file format from which data can be efficiently extracted (e.g., a spreadsheet rather than a PDF), including all relevant meta‐data listed in item 5. For any data inputs that cannot be shared because of ethical or legal reasons, such as third‐party ownership, provide a contact name or the name of the institution that retains the right to the data. | https://ghdx.healthdata.org/record/ihme-data/rhdei |
| **Data analysis** | | |
| **9** | Provide a conceptual overview of the data analysis method. A diagram may be helpful. | 4 |
| **10** | Provide a detailed description of all steps of the analysis, including mathematical formulae. This description should cover, as relevant, data cleaning, data pre‐processing, data adjustments and weighting of data sources, and mathematical or statistical model(s). | 4-7 |
| **11** | Describe how candidate models were evaluated and how the final model(s) were selected. | 7 |
| **12** | Provide the results of an evaluation of model performance, if done, as well as the results of any relevant sensitivity analysis. | 7, Supplement |
| **13** | Describe methods for calculating uncertainty of the estimates. State which sources of uncertainty were, and were not, accounted for in the uncertainty analysis. | N/A |
| **14** | State how analytic or statistical source code used to generate estimates can be accessed. | https://github.com/ihmeuw/rhdei |
| **Results and Discussion** | | |
| **15** | Provide published estimates in a file format from which data can be efficiently extracted. | https://ghdx.healthdata.org/record/ihme-data/rhdei |
| **16** | Report a quantitative measure of the uncertainty of the estimates (e.g. uncertainty intervals). | https://ghdx.healthdata.org/record/ihme-data/rhdei |
| **17** | Interpret results in light of existing evidence. If updating a previous set of estimates, describe the reasons for changes in estimates. | 10-12 |
| **18** | Discuss limitations of the estimates. Include a discussion of any modelling assumptions or  data limitations that affect interpretation of the estimates. | 12 |

# Table S2. Principal component analysis factor loadings for 1990 and 2021

| Variable | Year | Coefficients | | | | | |
| --- | --- | --- | --- | --- | --- | --- | --- |
|  |  | PC1 | PC2 | PC3 | PC4 | PC5 | PC6 |
| Socio-demographic Index | 1990 | -0.45 | 0.10 | -0.28 | 0.18 | -0.07 | 0.82 |
|  | 2021 | -0.44 | 0.04 | -0.12 | 0.49 | 0.23 | 0.71 |
| Healthcare Access and Quality Index | 1990 | -0.43 | 0.09 | -0.29 | 0.57 | 0.46 | -0.43 |
|  | 2021 | -0.42 | -0.26 | -0.45 | 0.14 | 0.46 | -0.57 |
| Sanitation Access​ | 1990 | -0.44 | 0.14 | -0.18 | -0.09 | -0.78 | -0.37 |
|  | 2021 | -0.41 | 0.47 | 0.16 | 0.41 | -0.53 | -0.36 |
| Water Access​ | 1990 | -0.42 | 0.23 | -0.05 | -0.77 | 0.41 | -0.07 |
|  | 2021 | -0.40 | 0.50 | 0.34 | -0.54 | 0.43 | 0.01 |
| Underweight​ | 1990 | -0.39 | 0.08 | 0.90 | 0.18 | -0.01 | 0.04 |
|  | 2021 | -0.36 | -0.66 | 0.65 | -0.01 | -0.10 | -0.03 |
| All Cause Death​ | 1990 | -0.29 | -0.95 | -0.02 | -0.11 | 0.02 | -0.02 |
|  | 2021 | -0.41 | -0.16 | -0.47 | -0.53 | -0.51 | 0.21 |
| Standard Deviation | 1990 | 2.14 | 0.83 | 0.59 | 0.45 | 0.35 | 0.25 |
|  | 2021 | 2.20 | 0.68 | 0.60 | 0.39 | 0.34 | 0.24 |
| Proportion of Variance | 1990 | 0.77 | 0.11 | 0.06 | 0.03 | 0.02 | 0.01 |
|  | 2021 | 0.81 | 0.08 | 0.06 | 0.03 | 0.02 | 0.01 |
| Cumulative Proportion of Variance | 1990 | 0.77 | 0.88 | 0.94 | 0.97 | 0.99 | 1.00 |
|  | 2021 | 0.81 | 0.89 | 0.94 | 0.97 | 0.99 | 1.00 |


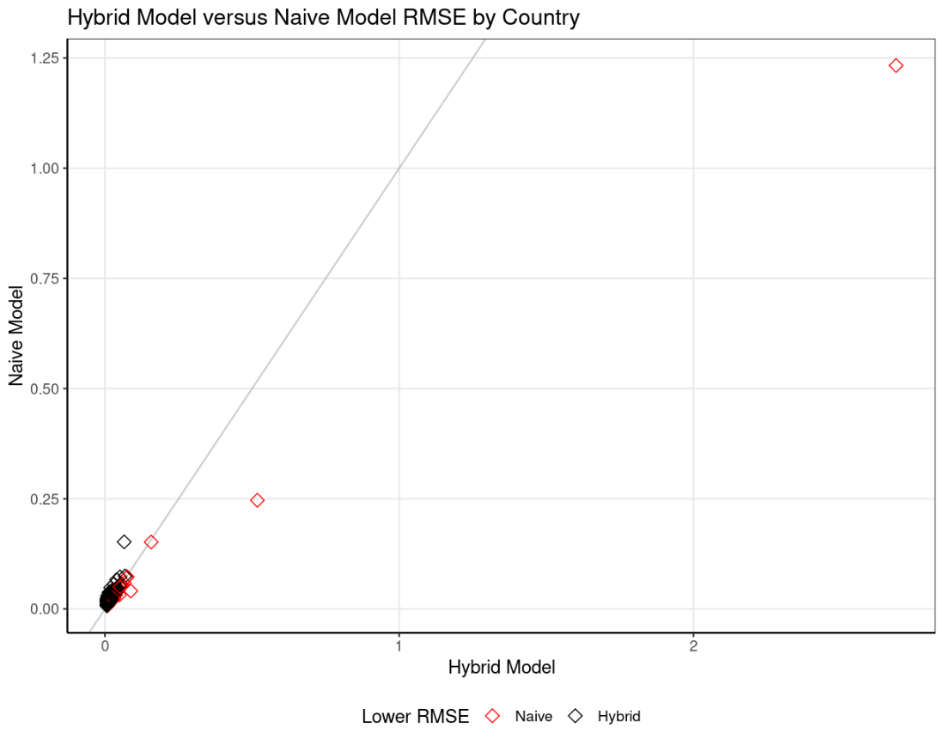


# Figure S2. Comparison of cross-validated model root mean square error (RMSE)
